# Integrating RNA-seq and scRNA-seq to explore the mechanism of macrophage ferroptosis associated with COPD

**DOI:** 10.3389/fphar.2023.1139137

**Published:** 2023-03-10

**Authors:** Pengbei Fan, Yige Zhang, Shenao Ding, Zhixin Du, Chunyu Zhou, Xiaodan Du

**Affiliations:** ^1^ School of Medicine, Henan University of Chinese Medicine, Zhengzhou, China; ^2^ The Second Clinical Medical College, Henan University of Chinese Medicine, Zhengzhou, China

**Keywords:** COPD, ScRNA-seq, molecular validation, macrophage, ferroptosis

## Abstract

**Aims:** Our study focused on whether macrophages ferroptosis is associated with the pathogenesis of chronic obstructive pulmonary disease (COPD) or not.

**Main methods:** We first identified macrophage module genes by weighted gene co-expression network analysis (WGCNA) in RNA sequencing (RNA-seq) date from COPD, and then identified macrophage marker genes by comprehensive analysis of single-cell RNA sequencing (scRNA-seq) data from COPD macrophages. There were 126 macrophage marker genes identified, and functional enrichment analyses indicated that ferroptosis pathway genes were significantly enriched. Secondly, we identified eight macrophage ferroptosis related genes and based on these eight genes, we performed co-expression analysis and drug prediction. Thirdly, two biomarkers (SOCS1 and HSPB1) were screened by the least absolute shrinkage and selection operator (LASSO), random forest (RF), and support vector machine-recursive feature elimination (SVM-RFE) and established an artificial neural network (ANN) for diagnosis. Subsequently, the biomarkers were validated in the dataset and validation set. These two biomarkers were then subjected to single gene-gene set enrichment analysis (GSEA) and gene set variation analysis (GSVA) analysis, and the ceRNA network was constructed. Finally, we carried out molecular validation with COPD models *in vitro* for cell counting kit-8 (CCK8) experiments, Western blot and quantitative real-time PCR (qRT-PCR) analysis and transmission electron microscopy (TEM).

**Key findings:** This study revealed the vital role of macrophage ferroptosis in COPD, and novel biomarkers (SOCS1 and HSPB1) may be involved in the pathogenesis of COPD by regulating macrophage ferroptosis.

**Significance:** Taken together, our results suggest that targeting SOCS1 and HSPB1 could treat COPD by inhibiting macrophage ferroptosis.

## 1 Introduction

As a major cause of morbidity, mortality, and healthcare use globally, the pathogenesis of chronic obstructive pulmonary disease (COPD) includes inflammation, oxidative stress, airway remodeling, and accelerated lung aging (Christenson et al., 2022; Yang et al., 2022). Characterized by chronic respiratory symptoms, COPD is an inflammatory lung condition (Wang et al., 2020). As the most abundant type of immune cell in the lung, macrophages are crucial to the inflammatory response associated with COPD (Higham et al., 2020). Several studies have demonstrated that macrophages contribute to the development of acute and chronic inflammatory responses through the secretion of inflammatory cytokines/chemokines and the activation of inflammation associated transcription factors (Singh et al., 2021; Li et al., 2022a). Furthermore, COPD patients have decreased macrophage clearance of apoptotic cells, resulting in persistent inflammation in the lungs (Grabiec and Hussell, 2016).

Death is the common fate for all life forms, from cells to organisms. In a healthy organism, cell death is an essential component of the development of the cell and the maintenance of internal environmental homeostasis, but when it is dysregulated, it can result in a variety of pathological consequences. Recently, researchers have proposed new types of death, including necroptosis, autophagic death, scorch death, and ferroptosis, based on different morphological characteristics of cell death (Han et al., 2020; Peng et al., 2022). Among these, ferroptosis, a form of regulated cell death discovered by Dixon et al., in 2012, has a significant influence on various diseases (Dixon et al., 2012). Several diseases have been linked to ferroptosis, such as tumors, neurodegenerative diseases, brain injuries, atherosclerosis, diabetes mellitus, and pulmonary inflammatory (Han et al., 2020; Sepand et al., 2021; Wang et al., 2021). Emerging evidence has revealed that pulmonary macrophage death plays important roles in the progression of inflammatory, while study shows ferroptosis strongly related to smoking and COPD progression (Liao et al., 2022). Moreover, pulmonary macrophage has a key role in the initiation and progression of the chronic inflammatory process in COPD lung tissue, a systematic understanding of macrophage ferroptosis involved in COPD is essential to explore key targets in COPD.

In recent years, single-cell RNA sequencing (scRNA-seq) has become a popular way to identify genes in various types of cells. In view of this advantage, we analyzed scRNA-seq and RNA sequencing (RNA-Seq) data from COPD macrophages and found that the pathogenesis of COPD is closely related to ferroptosis in macrophages. Afterwards, we identified ferroptosis related differential genes (DEGs) and pathways, screened for biomarkers, performed drug prediction, and constructed a ceRNA network. In addition, we studied further cellular experiments to verify the related gene expression, which will provide potential biomarkers and therapeutic targets for COPD.

## 2 Materials and methods

### 2.1 Acquisition of scRNA-seq and RNA-Seq data

The scRNA-seq data of COPD macrophages and RNA-Seq data used in this study were screened from the public database GEO database platform (http://www.ncbi.nlm.nih.gov/geo). The scRNA-seq dataset (GSE183974) used is based on the Illumina NovaSeq assay platform and contains 9 samples. And the GSE13896 and GSE130928 were used as the dataset and validation set, respectively. GSE13896 included 12 COPD patients and 24 controls, while GSE130928 included 22 COPD patients and 24 controls.

### 2.2 Immune infiltration analysis

CIBERSORT inverse convolution estimates 22 immune cell types within tissues using linear support vector regression. Initially, CIBERSORT was used to calculate the relative abundance of immune cell types in samples. Using the Wilcoxon test, immune cells were compared among COPD samples and control samples. Lastly, R software was used to calculate correlation coefficients between different immune cells based on the results obtained through the CIBERSORT inverse convolution method.

### 2.3 Identifying macrophage-related modules

The weighted gene co-expression network analysis (WGCNA) technology is a high-throughput algorithm for analyzing gene expression data. Initially, we selected a soft threshold for constructing an adjacency matrix. The adjacency matrix was then converted to the topological overlap matrix (TOM) and the corresponding dissimilarity (1-TOM). In the following steps, a hierarchical clustering dendrogram was established to group genes with similar expression profiles into modules. Using the merged modules, associations were identified with immune cells, the module-immune cell correlation heat map was plotted and the module with the highest correlation with macrophages were screened.

### 2.4 Analysis of scRNA-seq data

The scRNA-seq data was converted into Seurat objects with the help of the R software “Seurat package”. Following quality control and data filtering, the top 1500 highly variable genes were analyzed using principal component analysis (PCA) to identify the 12 most significant principal components (PC). Then, using t-distributed stochastic neighbor embedding (t-SNE), we were able to achieve unsupervised clustering and unbiased visualization of cell subpopulations. The gene expression differences between clusters were compared using the “FindAllMarkers” package. To identify the marker genes for each cluster, |log2 FC (fold change)| > 1 and adjusted *p* < 0.05 were used. Additionally, subpopulations within each cluster were annotated using the “SingleR” package.

We drew a venn diagram by intersecting macrophage marker genes with macrophage module genes. Afterwards, the intersecting genes were analyzed for functional enrichment. Gene ontology (GO) and Kyoto encyclopedia of genes and genomes (KEGG) pathway enrichment analysis was done using the R package “clusterProfiler”, and the q-value and *p*-value cutoffs were set to 0.05. The results of the enrichment analysis were visualized using R software.

### 2.5 Identification and enrichment analysis of macrophages ferroptosis-related genes

To explore potential ferroptosis-related genes in macrophages, we intersected macrophage marker genes with ferroptosis-related genes obtained from FerrDb (http://www.zhounan.org/ferrdb/) and drew a Venn diagram, after which the obtained macrophage ferroptosis-related genes were subjected to functional enrichment analysis by R software.

### 2.6 Drug prediction of macrophage ferroptosis-related genes

To identify drugs that target genes, the Drug-Gene Interaction Database (DGIdb, http://dgidb.genome.wustl.edu/) be used. Based on the DGIdb database, we were able to identify potential drug candidates targeting macrophage ferroptosis-related genes. And the network of drug-genes was plotted using Cytoscape software.

### 2.7 Determination of macrophage ferroptosis-related DEGs

To begin with, DEGs were identified using the “limma” package and were defined according to the screening criteria (|log2FC| > 1, *p < 0.05*). The heatmap and volcano plot of DEGs were constructed using the R packages “Pheatmap” and “ggplot2”. We then conducted a Pearson’s correlation analysis on DEGs and macrophage ferroptosis-related genes to identify DEGs that may be associated with macrophage ferroptosis. Our thresholds were as follows: correlation coefficient (|r|) > 0.5 and *p < 0.001.* Functional enrichment analysis was conducted on these macrophage ferroptosis-related DEGs.

### 2.8 Screening of biomarkers using machine learning techniques

Machine learning is the new tool for analyzing algorithms. The least absolute shrinkage and selection operator (LASSO) is a regularized regression algorithm using the “glmnet” package. Using Support vector machine-recursive feature elimination (SVM-RFE), features can be ranked based on recursion. Random forest (RF) analysis allows feature selection, calculates the mean decrease gini (MDG) for each gene and ranks them, and determines the optimal number of features by adding differential genes one by one from the largest to the smallest MDG value to determine the accuracy of the classification results. The three algorithms were used to better screen for biomarkers.

### 2.9 Artificial neural network construction

Using the packages “neuralnet” and “neuralnettools” in R software, we constructed an artificial neural network (ANN) model for the biomarkers based on the gene score. The number of hidden nodes was set to five. Based on the derived “gene score” information, a classification model for COPD was constructed. To evaluate the predictive performance of the ANN, the receiver operating characteristic curves (ROC) were used both on the dataset and on the validation set.

### 2.10 ROC analysis and validation of the biomarkers

In addition, the gene expression and diagnostic value of biomarkers was verified in both GSE13896 and GSE130928. We examined the diagnostic effectiveness of the biomarkers with the ROC using the “pROC” package. The expression levels of the biomarkers were also compared between COPD and control samples using an independent *t*-test, with a *p < 0.05* considered statistically significant.

### 2.11 GSVA and GSEA analyses

According to the median expression levels of biomarkers in the GSE13896 dataset, COPD samples were divided into two groups (high and low-expression group). And single gene-gene set variation analysis (GSVA) and gene set enrichment analysis (GSEA) analyses were used to clarify the enriched KEGG pathways, using the gene set “c2. cp.kegg.v7.4. symbols.gmt” as a reference. Gene sets with *p < 0.05* were treated as significantly enriched gene sets.

### 2.12 ceRNA network construction

To explore the ceRNA network, the miRNet (https://www.mirnet.ca/miRNet/home.xhtml) were performed to identify possible miRNA targeting biomarkers. Only the intersection of miRNAs targeting biomarkers from databases were selected for ceRNA network construction. Based on data from the ENCORI (http://starbase.sysu.edu.cn/index.php), we identified lncRNAs that target co-miRNAs. The ceRNA regulation network was then constructed. Cytoscape was used to visualize the network.

### 2.13 Cell lines and agents

Human monocyte leukemia cell line THP-1 were obtained from the Procell Life Science and Technology Collection (Procell, Wuhan, China) and cultured with RPMI1640 medium (Solarbio, Beijing, China) supplemented by 10% fetal bovine serum (Gibco, Australia) in 37°C, 5% CO2 humid incubator. 12-O-tetradecanoyl phorbol-13-acetate (PMA) was purchased from Glpbio, storage at 4°. THP-1 was induced by 100 ng/mL PMA for 24 h prior to the experiment to adhere and differentiate into macrophages.

Smoking is one of the major causes of COPD. In our experiment, we used cigarette smoke extract (CSE) to stimulate macrophages *in vitro*, to simulate macrophages of COPD patients. CSE was prepared as described. Briefly, three cigarettes (Yuxi, Hongta Tobacco Co., China) were ignition and combustion and the smoke was collected using a vacuum pump through a container containing the phosphate-buffered saline (PBS, 15 mL) maker as 100% CSE. This 100% CSE was adjusted to pH 7.4 and aseptically filtered through a 0.22-µm filter. CSE was prepared fresh for each experiment and diluted with medium containing 10% FBS immediately prior to use.

### 2.14 Cell viability

THP-1 were planted with 6000 cells per well in 96-well plates and PMA induced macrophages for 24 h, then treated with several concentration of 2.5%, 5%, 10% CSE for 12 h, 24 h, 48 h. After that, we used the cell counting kit-8 (CCK8; APExBIO, America) to measure cell viability. Refer to the manufacture’s instruction and add 10 μL/well CCK8 reagent to the cells. After incubated at 37°C for 3 h, incubated for 30min at 37°C. The Opticaldensity (OD) value was measured at 450 nm using a Microplate Reader (Bio-Tek, Vermont, United States).

### 2.15 Western blotting

THP-1 were planted in 6-well plates with 2*10^5^ cells per well and PMA induced macrophages for 24 h, then treated with specified concentration of 5% CSE or PBS for 12 h, 24 h, 48 h. The cells were washed with PBS for three times. Collecting cells in each group by trypsin digestion, adding RIPA (Servicebio, G2002-100 ML) lysis buffer containing PMSF (Servicebio, G2008-1 Ml), collecting that supernatant to obtain total protein solution, then protein denaturation. After samples were separated by 12% SDS-PAGE gel electrophoresis, transferred to PVDF membranes (Servicebio, WGPVDF45), blocked with 5% defatted milk for 1 h, the membrane was incubated overnight with primary antibodys (1:1000 diluted GPX4, Servicebio, GB113091; 1:5000 diluted GAPDH, Proteintech, 6004–1) at 4°C, followed by TBST washing three times the next day, incubation with secondary antibody (1:20,000 diluted) for 1 h. Following incubation and washing, uniformly dropwise added that high-sensitivity ECL luminescent liquid (Servicebio, G2014-50 ML). The bands were detected using the Bio-RadChemiDoc MP luminescence imaging system. The images in the film were quantified for the gray value using ImageJ.

### 2.16 Determination of GPX4, SOCS1 and HSPB1 secretion in macrophages

THP-1 were planted in 6-well plates with 2*10^5^ cells per well and PMA induced macrophages for 24 h, then treated with specified concentration of 5% CSE or PBS for 12 h, 24 h, 48 h. These total RNA was extracted from 5% CSE-induced macrophages as a COPD model (CSE_12h_, CSE_24h_, CSE_48h_) and 5% PBS-induced macrophages as a control group (Con), using RNA-Quick Purification Kit (EScience, Shanghai, China) and quantified by Nanodrop spectrophotometer (NanoDrop 2000, Thermo, United States). Complementary cDNA was synthesized from total RNA using the Servicebio®RT First Strand cDNA Synthesis Kit (Servicebio, Wuhan, China). The qRT-PCR assay was performed using the SYBR Green qPCR Master Mix (None ROX) (Servicebio, Wuhan, China) on Quant Studio Real-Time PCR System (Thermo Fisher Scientific, United States). Determination of GPX4, SOCS1 and HSPB1 secretion were normalized to GAPDH and calculated with the 2^−ΔΔCT^ method. Three biotechnical replicates were set up for each sample. The primer sequences used in this study see in Table 1.

**TABLE 1:** The primer sets for associated mRNA.

Human	Forward primer (5′-3′)	Reverse primer	Fragment length (bp)
GPX4	TGA​AGA​TCC​AAC​CCA​AGG​GC	GAC​GGT​GTC​CAA​ACT​TGG​TG	75
SOCS1	GAC​ACG​CAC​TTC​CGC​ACA​TT	TAG​AAT​CCG​CAG​GCG​TCC​A	86
HSPB1	AGC​ATG​GCT​ACA​TCT​CCC​GG	GAC​TCG​AAG​GTG​ACT​GGG​ATG	172
GAPDH	GGA​AGC​TTG​TCA​TCA​ATG​GAA​ATC	TGA​TGA​CCC​TTT​TGG​CTC​CC	168

### 2.17 Transmission electron microscopy

THP-1 were planted in 6-well plates with 2*10^5^ cells per well and PMA induced macrophages for 24 h, then treated with the concentration of 5% CSE or PBS for 48 h. Macrophages were stimulated with 5% CSE or PBS for 48 h. Macrophages were gently scraped from the culture dish using a cell scraper, and the cells were collected by centrifugation in the 1.5 ml Eppendorf tubes. And medium was removed and pre-chilled 2.5% glutaraldehyde (pH 7.4) solution was added for 2 h at 4°C, resuspended and fixed at 4 °C. Then macrophages were fixed and stored at 4°C. Cells or bacteria were centrifuged by centrifuge, supernatant was discarded and 0.1 M phosphate buffer (pH 7.4) was added, mixed and rinsed for 3 min before centrifugation, and the washing was repeated 3 times. Prepare 1% agarose solution by heating and dissolving in advance, add to EP tubes after cooling slightly, and pick up the precipitate with forceps before the agarose solidifies and wrap it in agarose in suspension. The specimens were sequentially dehydrated using 30% alcohol −50% alcohol −70% alcohol −80% alcohol −95% alcohol −100% alcohol −100% alcohol for every 20 min then 100% acetone twice for every 15 min. Finally, after permeabilization, embedding, and sectioning, the samples were observed under transmission electron microscopy and images were collected for analysis.

## 3 Results

### 3.1 Identification of macrophage module genes

**FIGURE 1:**
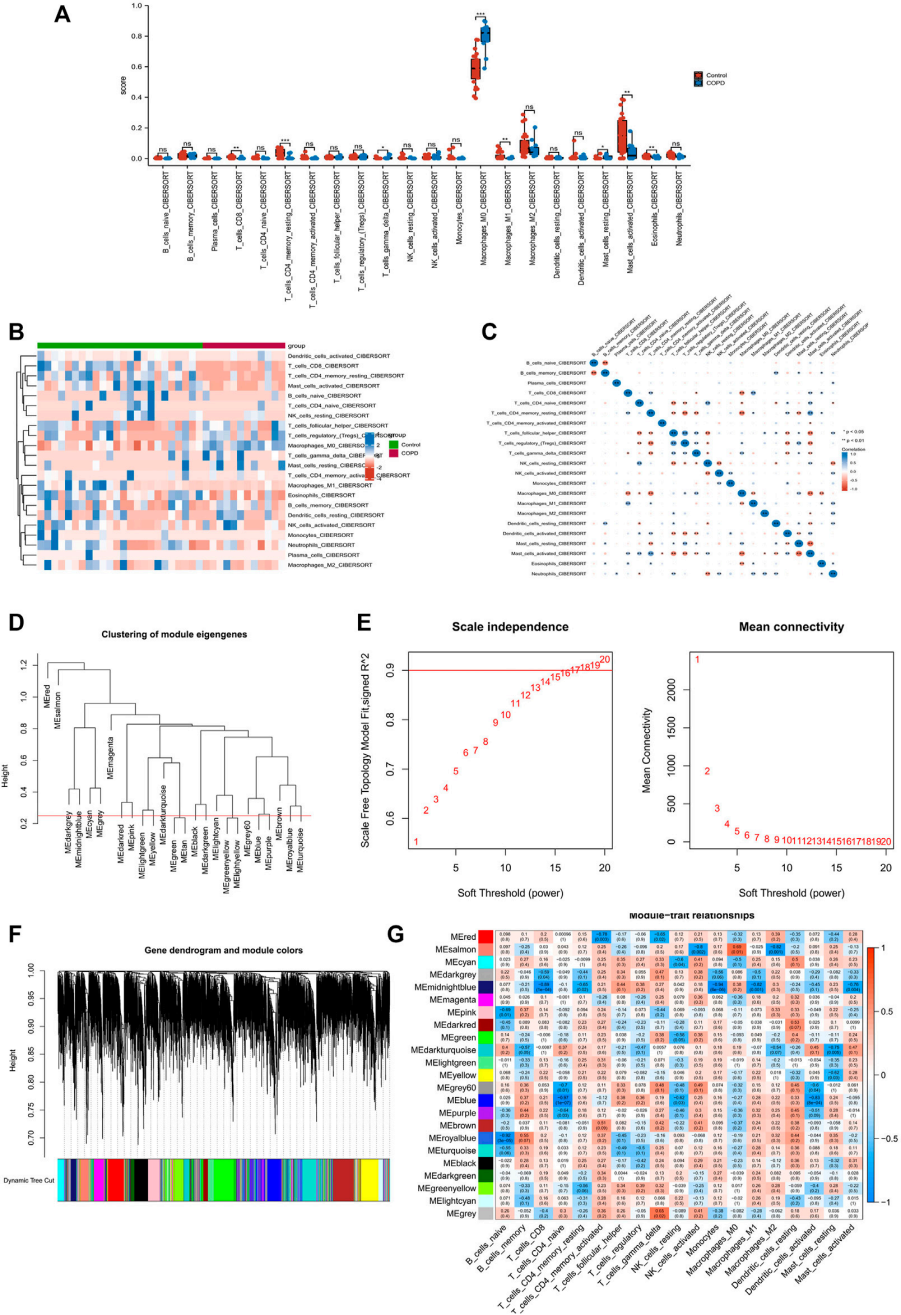
Immune infiltration analysis and identification of macrophage-related modular genes. **(A)** The CIBERSORT algorithm was used to analyze the content of immune cells. **(B)** A holistic view of the distribution of immune cells between different tissues. **(C)** Correlation analysis was performed on all immune cells in the CIBERSORT algorithm. **(D)** Correlation diagram between modules obtained by clustering according to inter-gene expression levels. **(E)** Screening for suitable soft thresholds and scale-free network validation. The soft threshold is selected as 16. **(F)** The cluster dendrogram with the gene modules and module merging. **(G)** The correlation between gene modules and immune cell fraction.

The results of CIBERSORT showed that both M0 macrophage and M1 macrophages were altered in the COPD group (Figure 1A). Figure 1B illustrates the immune cell heat map of COPD, and Figure 1C illustrates the correlation analysis, which showed a remarkable correlation in the 22 types of immune cells. Following this, we performed the WGCNA analysis. As a first step, we set the threshold at 0.25 to merge similar modules (Figure 1D). Then, we chose *β* = 16 as a suitable soft threshold for the construction of a scale-free network (Figure 1E). After merging similar gene modules, a dynamic cut tree was generated (Figure 1G). Of the 23 gene modules, the midnight blue module shows a close relationship with the features of M1 macrophages and M0 macrophages (Figure 1H). In the end, we obtained 763 macrophage module genes.

### 3.2 Identification of macrophage marker genes and functional enrichment analysis

**FIGURE 2:**
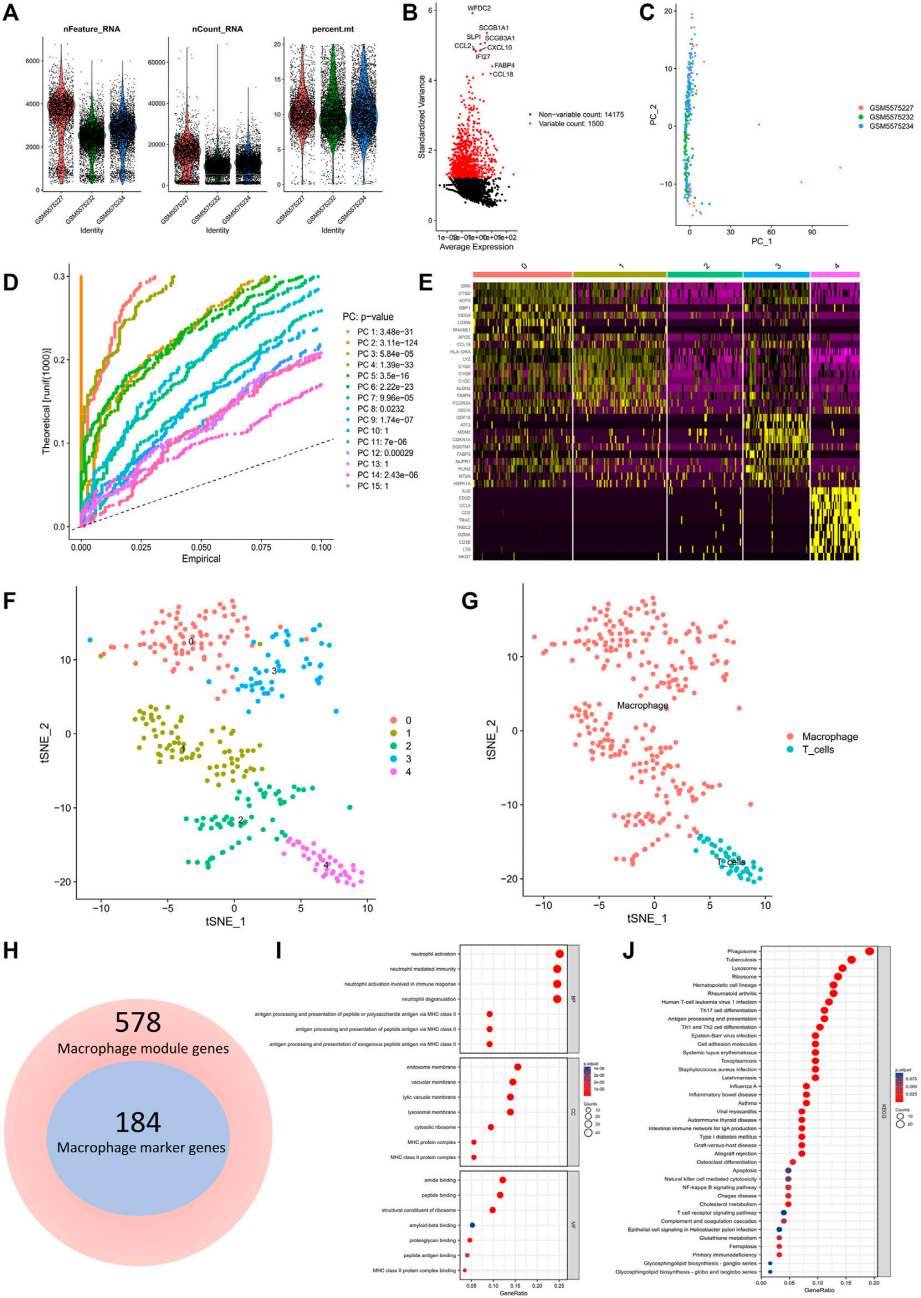
Identification of macrophage cell marker genes by scRNA-seq analysis. **(A)** Quality control of scRNA-seq data. **(B)** The variance plot showed 14,175 genes in all cells, red dots represent the top 1500 highly variable genes. **(C)** PCA was used for dimensionality reduction. **(D)** 12 PCs were identified based on *p < 0.05*. **(E)** The heatmap showed the relative expression of genes in 5 clusters. Yellow represents high expressed genes and purple represents low expressed genes. **(F)** 5 clusters were visualized based on the t-SNE algorithm. **(G)** Cell subpopulations identified by marker genes. **(H)** Venn diagram illustrating macrophage module genes and macrophage marker genes. **(I,J)** GO and KEGG analysis of macrophage marker genes.

Figure 2A shows the range of gene numbers detected, the depth of sequencing, and the percentage of mitochondrial content in each sample. Following quality control and normalization of the data, we selected the top 1500 genes with high variability (Figure 2B). The PCA method was used to reduce dimensionality (Figure 2C), and 12 PCs with *p < 0.05* were selected for further analysis (Figure 2D). We identified 5 clusters using the t-SNE algorithm. The heatmap illustrating the relative expression of five cluster marker genes (Figure 2E). Following this, we visualized the five clusters (Figure 2F). The “singleR” algorithm was used to annotate cell subpopulations, and clusters 0, 1, 2, 3 and 4 were identified as macrophage subpopulations (Figure 2G). Finally, we identified 184 macrophage marker genes.

Interestingly, we found that the macrophage marker genes completely overlapped with the macrophage module genes (Figure 2H). After that, GO analysis showed that the biological processes (BP) were mainly focused on neutrophil activation, neutrophil-mediated immunity and neutrophil activation involved in immune response. Cell composition (CC) was mainly focused on endoplasmic membrane, vacuolar membrane, vesicular membrane and lysosomal membrane and molecular functions (MF) were mainly focused on amide binding, peptide binding, amyloid-beta binding and proteoglycan binding. The KEGG signaling pathway was significantly enriched in cell adhesion molecules, NF-κB signaling pathway, apoptosis and ferroptosis. Ferroptosis, as a pathway enriched by COPD macrophage marker genes, is likely to play a great role in the pathology of COPD, therefore, we selected ferroptosis-related genes for the next study.

### 3.3 Identification and functional enrichment analysis of macrophage ferroptosis-related genes

**FIGURE 3:**
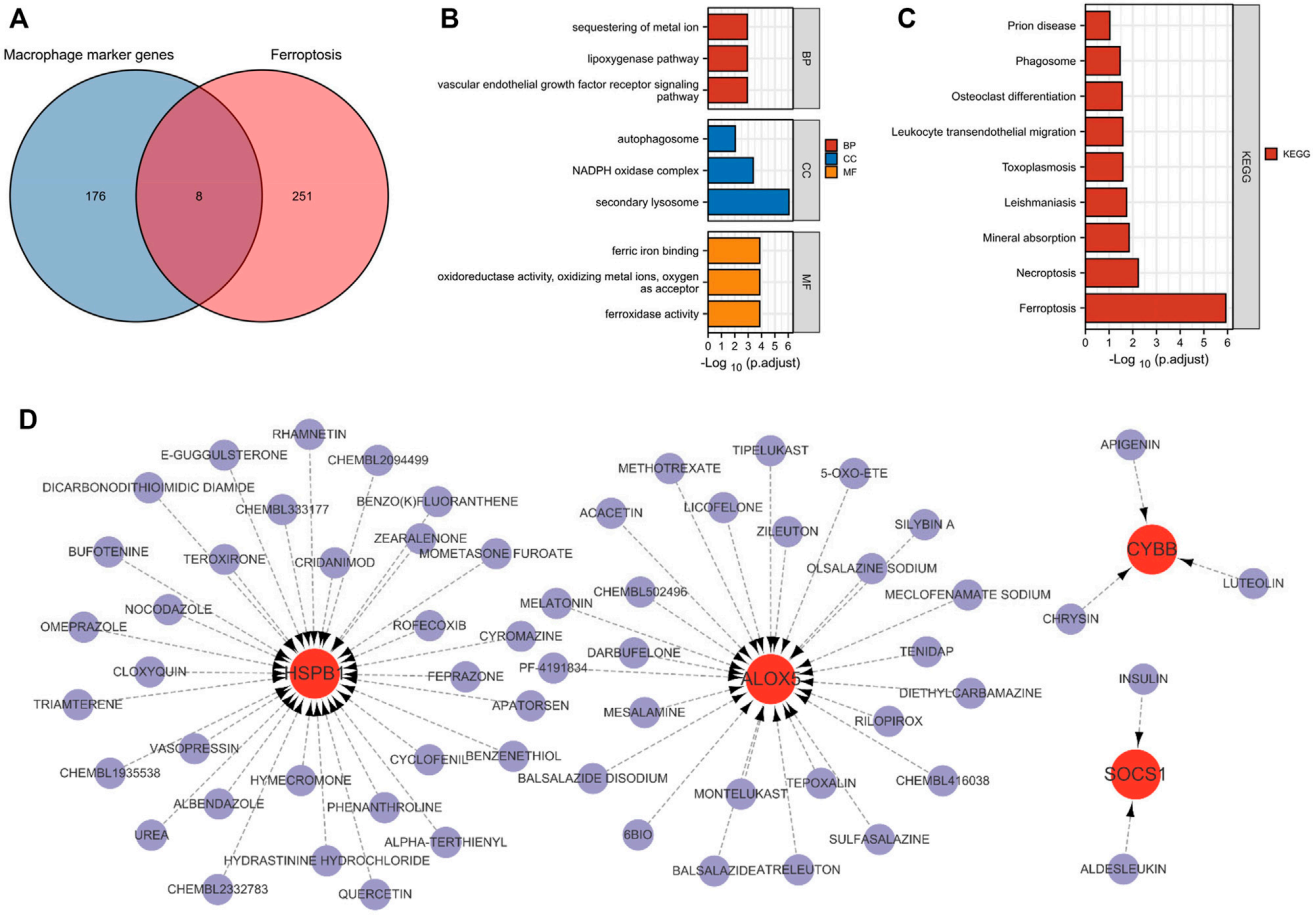
Macrophage ferroptosis-related genes identification and drug prediction. **(A)** Venn plot exhibiting the macrophage marker genes and ferroptosis-related genes. **(B,C)** GO and KEGG analysis of Macrophage ferroptosis-related genes. **(D)** The drug and macrophage ferroptosis-related genes interaction network.

A total of 8 macrophage ferroptosis-related genes were identified (Figure 3A), and further functional enrichment analysis revealed that GO analysis (Figure 3B) showed that the biological processes were mainly focused on the lipoxygenase pathway and vascular endothelial growth factor receptor signaling pathway. The cellular composition was mainly focused on autophagosomes, NADPH oxidase complex, secondary cell bodies. The molecular functions were mainly focused on iron binding, and KEGG analysis (Figure 3C) showed that Leukocyte trans endothelial migration, necroptosis and ferroptosis signaling pathways were significantly enriched.

### 3.4 Identification of the potential drugs

A total 61 compounds or drugs corresponding to genes were identified. As shown in Figure 3D, two drugs target SOCS1, 31 drugs target HSPB1, 25 drugs target ALOX5, and three drugs target CYBB. Among them, insulin (targeting SOCS1), quercetin (targeting HSPB1), zileuton (targeting ALOX5) and apigenin (targeting CYBB) could be the potential effective drugs in the future.

### 3.5 Identification and functional enrichment analysis of macrophage ferroptosis‐related DEGs

**FIGURE 4:**
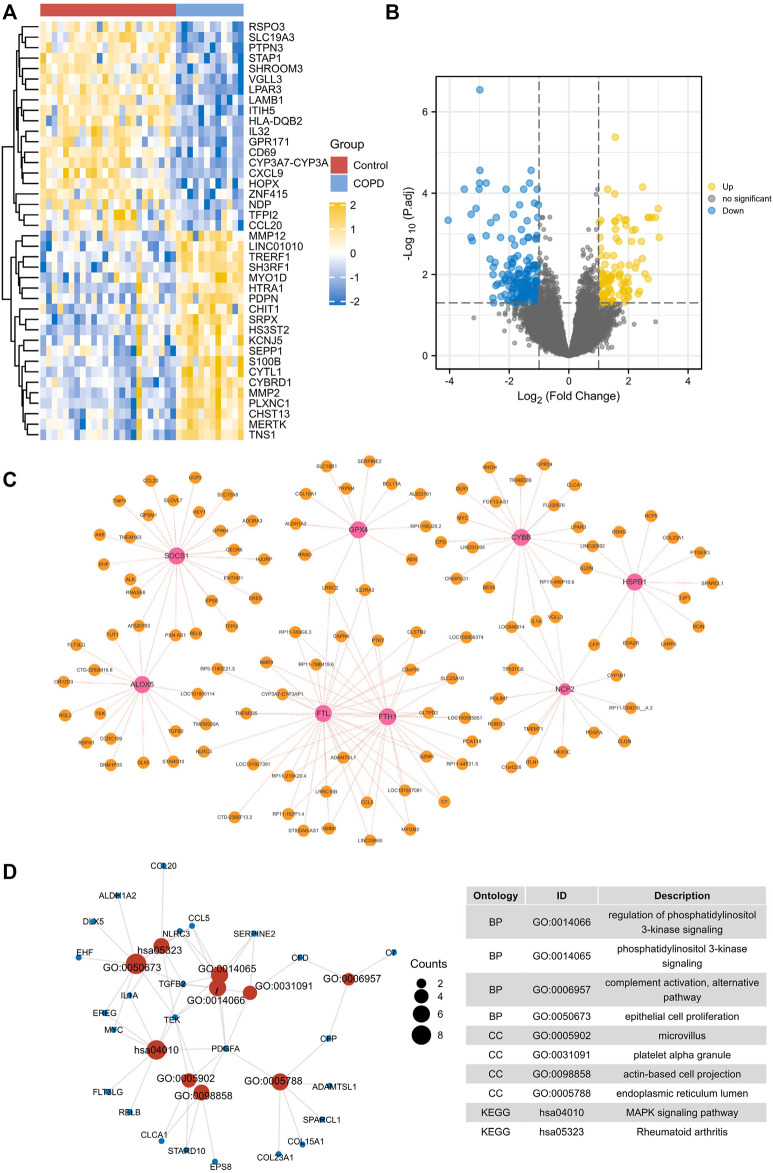
Identification and enrichment analysis of macrophage ferroptosis-related DEGs. **(A,B)** heat maps and volcano maps of GSE13896. **(C)** Macrophage ferroptosis related genes and DEGs interaction network. **(D)** GO and KEGG pathways enriched by the macrophage ferroptosis-related DEGs.

Next, we aimed to identify macrophage ferroptosis-elated DEGs. First, 845 DEGs were identified, and heat maps and volcano maps are shown in Figures 4A,B. In total, 121 macrophage ferroptosis-related DEGs were identified by correlation analysis (Figure 4C). According to functional enrichment analysis (Figure 4D), macrophage ferroptosis-related DEGs are functionally enriched in the regulation of phosphatidylinositol 3-kinase signaling, phosphatidylinositol 3-kinase signaling and MAPK signaling pathway.

### 3.6 Construction of biomarkers

**FIGURE 5:**
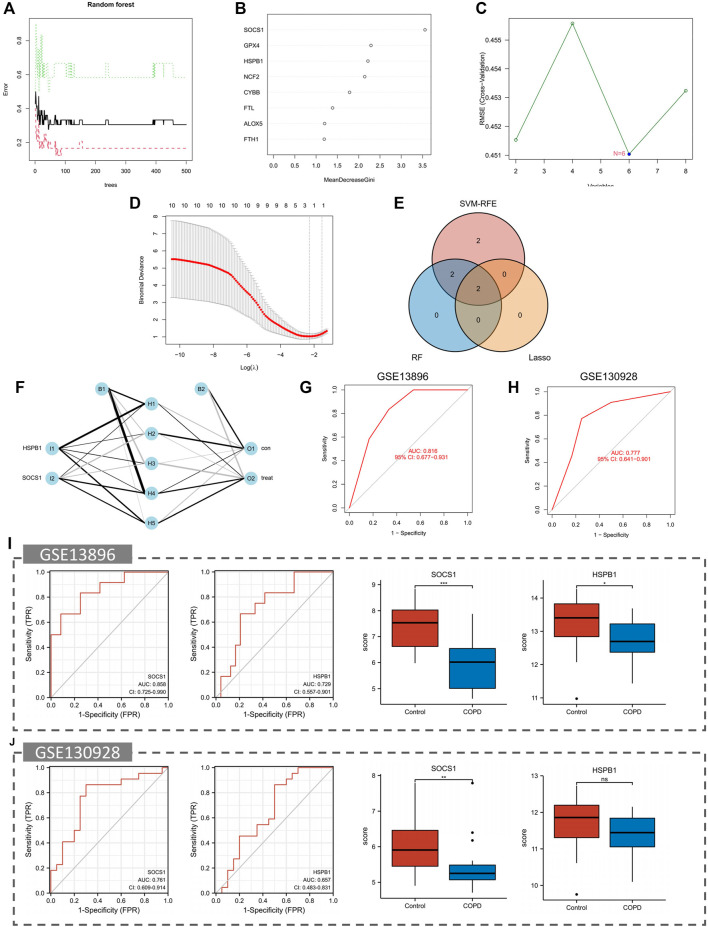
Identification and validation of the biomarkers. **(A,B)** RF algorithm. **(C)** SVM-RFE algorithm. **(D)** LASSO regression analysis. **(E)** Venn plot exhibiting the reliable biomarkers among LASSO, SVM-RFE, and RF. **(F)** The visualization of the ANN diagnostic model. **(G)** The assessment result of the date set. **(H)** The testification result of the validation set. **(I)** Validation of the key crosstalk genes in GSE13896. **(J)** Validation of the key crosstalk genes in GSE130928.

Four diagnostic genes were identified by the RF algorithm (Figures 5A, B). By SVM-RFE algorithm, six genes were extracted as candidate biomarkers (Figure 5C). Using the LASSO regression algorithm, two genes were identified as potential diagnostic biomarkers (Figure 5D). Using a Venn diagram, two genes (SOCS1 and HSPB1) were then overlapping and served as robust diagnostic biomarkers.

### 3.7 Development and validation of an ANN model

Based on gene weight, an ANN diagnostic model was developed (Figure 5F). During testing of the model, the AUC was 0.816 for the date set and 0.777 for the validation set, indicating that the model performed very well in diagnosing COPD. Results showed that we had developed a good diagnostic model between COPD samples and controls.

### 3.8 ROC analysis and validation of the biomarkers

We validated the biomarkers in GSE13896 (Figure 5I) and GSE130928 (Figure 5J) to further verify their efficacy. According to the ROC results in the GSE13896 dataset, HSPB1 and SOCS1 were effective at discriminating between COPD and control samples. Furthermore, the expression of the biomarkers was significantly lower in COPD samples, suggesting that HSPB1 and SOCS1 are strongly associated with COPD. In addition, the ROC results showed good diagnostic efficacy in the validation dataset, and the levels of mRNA expression of the biomarkers were lower in case groups.

### 3.9 GSVA and GSEA analysis

**FIGURE 6:**
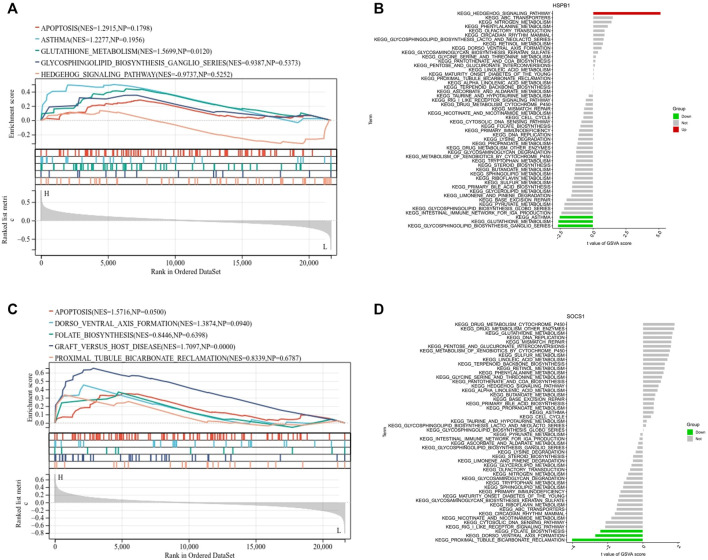
Single gene GSEA and GSVA analysis. **(A,B)** GSEA and GSVA analysis of HSPB1. **(C,D)** GSEA and GSVA analysis of SOCS1.

GSEA analysis showed significant differences between different expression groups of HSPB1 in autophagy, asthma, and glutathione metabolism signaling pathways (Figure 6A), and different expression groups of SOCS1 in autophagy and folate biosynthesis signaling pathways, *etc.* (Figure 6C). After that, GSVA was used to analyze the relevant signaling pathways regulated by biomarkers. The results showed that the Hedgehog signaling pathway was highly enriched in the high expression group of HSPB1, and the low expression of HSPB1 was mainly enriched in asthma and glutathione metabolic signaling pathway (Figure 6B); the low expression of SOCS1 was mainly enriched in folate biosynthesis signaling pathway and so on (Figure 6D).

### 3.10 Construction of ceRNA network

**FIGURE 7:**
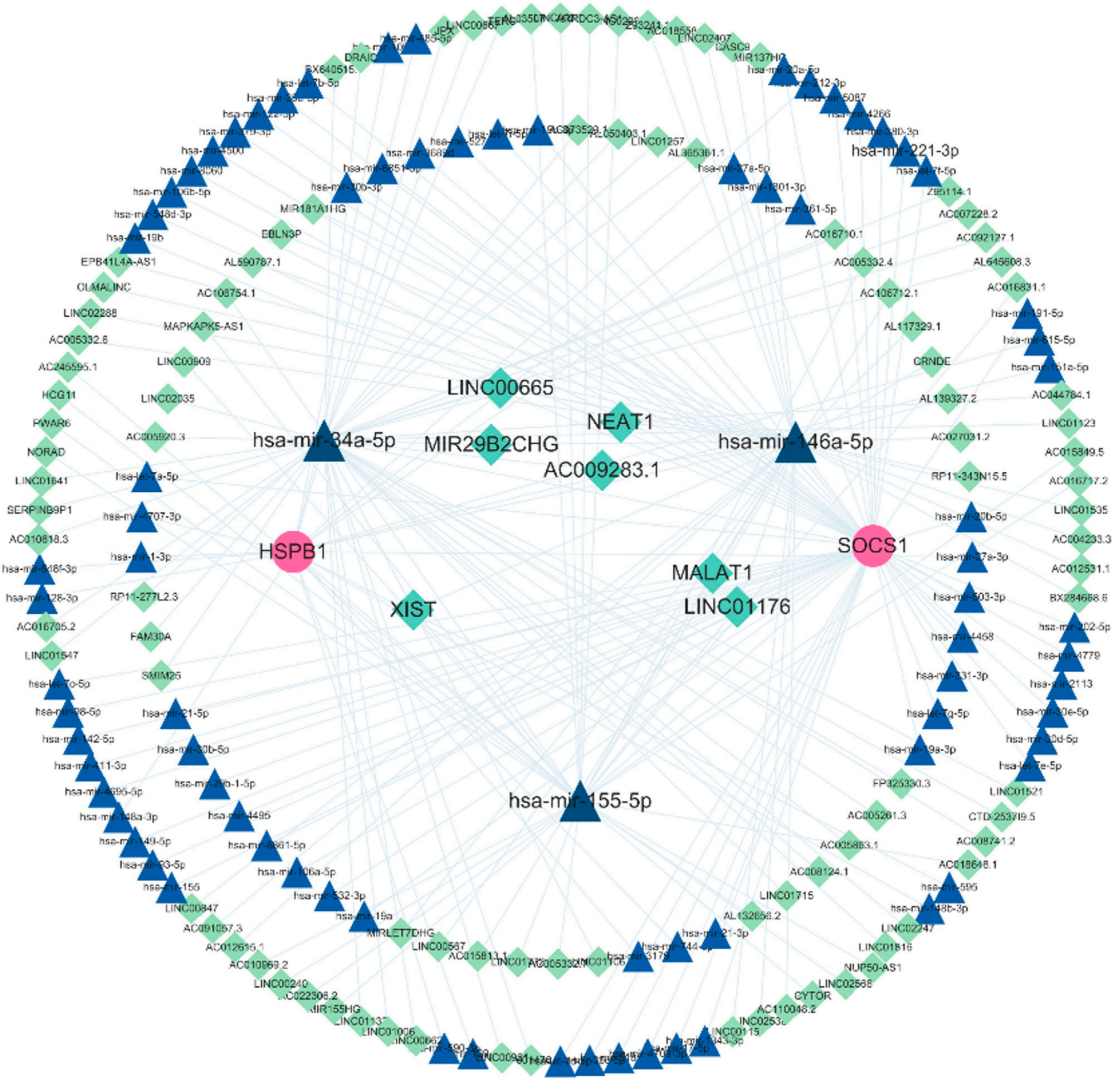
The ceRNA network. The ceRNA network was constructed through Cytoscape software. Triangles represent miRNAs, circles represent biomarkers, and lozenges represent lncRNAs.

The miRNet database was used to extract the target miRNAs of the 2 biomarkers of macrophage ferroptosis, and a total of 84 miRNAs were obtained, among which hsa-mir-146a-5p, hsa-mir-155–5p and hsa-mir-34a-5p were the co-miRNAs of the 2 biomarkers. After that, the 3 shared target miRNAs were entered into the ENCORI database, 114 target lncRNAs were obtained (Figure 7), among which, only XIST could target the 3 co-miRNAs simultaneously.

### 3.11 Cellular experimental validation

**FIGURE 8:**
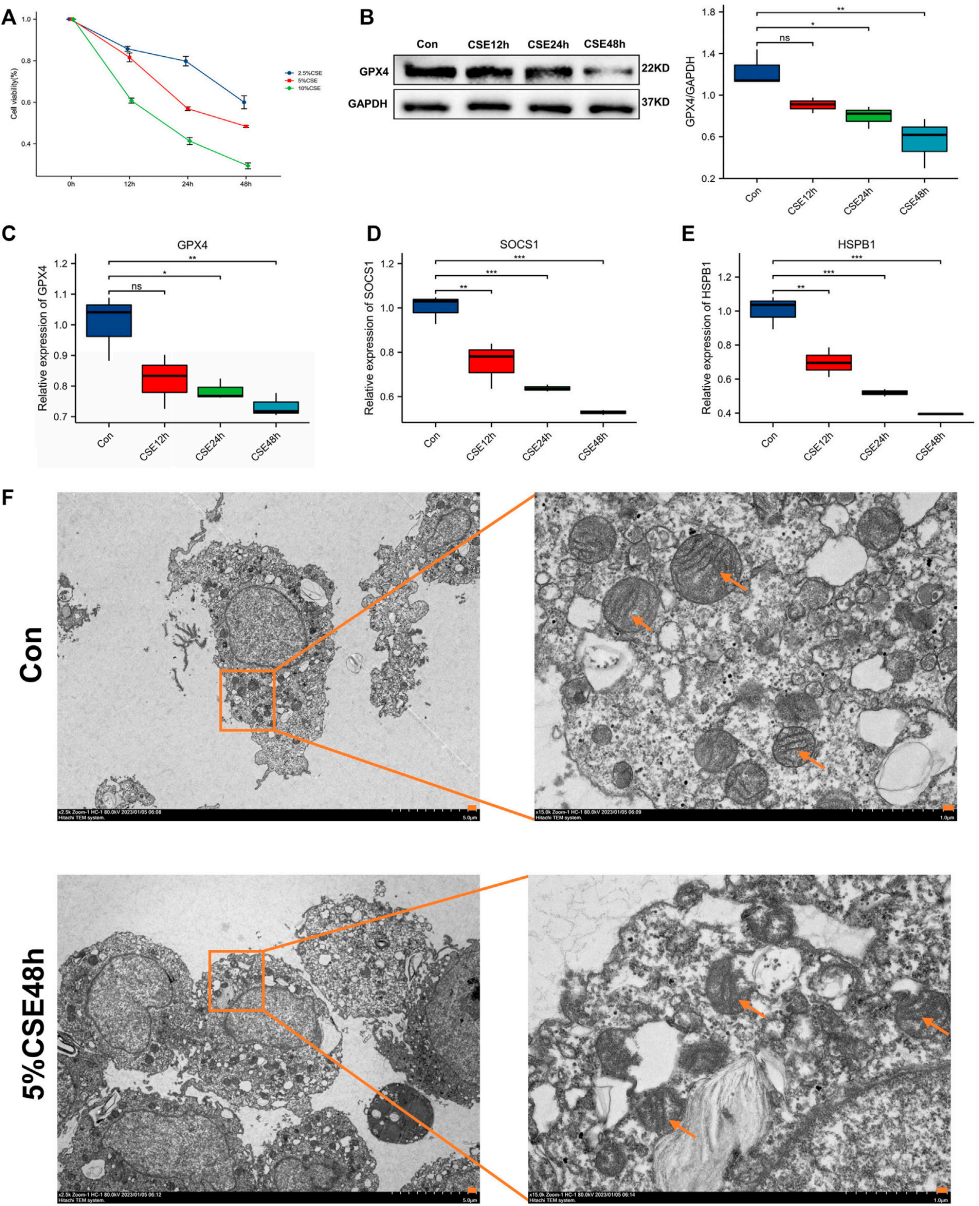
Cell viability and molecular validation. **(A)** Cell viability of macrophages stimulated by different concentrations of CSE. **(B)** Western blot for probing the expression of GPX4. **(C)** GPX4 expression after CSE stimulation at different time. **(D)** HSPB1 expression after CSE stimulation at different time. **(E)** SOCS1 expression after CSE stimulation at different time. *p < 0.05, **p < 0.01, ***p < 0.001, the CSE-induced group (CSE group) vs. the control group (Con). **(F)** Observation of mitochondrial morphology (orange arrows) using TEM.

Cellular experimental validation revealed no significant effect of CSE at 2.5%, 5% on macrophage viability at 12 h, with large changes in cell viability, and at 24 h and 48 h, CSE (5% concentration) significantly reduced macrophage viability, but the effect on viability was <50%, compared to 10% CSE which had >50% effect on cell viability (Figure 8A). Therefore, we used 5% CSE in the subsequent experiments. GPX4 played a vital role in ferroptosis. Though cellular experimental validation, we detected that GPX4 decreased in COPD macrophage (Figures 8B,C). This validates the relevance of macrophage ferroptosis to COPD. Furthermore, our results showed that the biomarkers HSPB1 and SOCS1 were significantly reduced in macrophages with increasing duration of CSE stimulation (Figures 8D,E). One of the most prominent morphological features of ferroptosis is mitochondrial changes. Compared with the control group, mitochondria are wrinkled, cristae are reduced, and membrane density is increased in CSE-induced 48 h group (CSE group), showing a typical expression of ferroptosis (Figure 8F).

## 4 Discussion

The high prevalence and mortality of COPD impose a heavy economic burden on society and families, with high annual direct medical costs, direct non-medical costs and indirect costs, and there are no effective measures to reverse the continuous decline of lung function in COPD patients (Adeloye et al., 2022). Due to the heterogeneity of COPD pathological mechanisms, its treatment is very challenging. Research has shown that macrophages play a significant role in the remodeling of small airways and chronic inflammation associated with this disease (Kaku et al., 2014; Lee et al., 2021). Genes associated with macrophages and their biological functions may provide insight into the molecular mechanisms underlying COPD. Therefore, the novel biomarkers identification of macrophages based on macrophage RNA-seq and scRNA-seq data is one of the key tasks to refine personalized and targeted drug use in COPD in the future.

In this study, we integrated scRNA-seq data of macrophages in COPD alveolar lavage fluid as well as RNA-Seq data and identified a total of 126 macrophage marker genes. Functional enrichment suggests that macrophage marker genes are mainly enriched in ferroptosis-related pathways. Macrophage ferroptosis has been found to play a vital role in lung disease, but no study has yet explored the role of macrophage ferroptosis in COPD. A total of eight COPD macrophage ferroptosis-related genes were identified in this study, including ALOX5, NCF2, CYBB, HSPB1, GPX4, FTH1, FTL, SOCS1. Researchers found that ALOX5 was significantly upregulated in macrophages of COPD mice, which led to lipid peroxidation and ferroptosis in alveolar macrophages, thereby exacerbating COPD (Gunes Gunsel et al., 2022). NCF2 is a key transcription factor in the antioxidant response, and can active the nicotinamide adenine dinucleotide phosphate oxidase (NOXs), then ferroptosis could be triggered (Li et al., 2022a). CYBB, also referred to as NOX2, is mainly expressed in lung macrophages (Seimetz et al., 2020). And NOX2 protein expression is found to be upregulated in the lungs of COPD mice and is involved in the oxidative and inflammatory response in the early stages of COPD (Zhang et al., 2022). Heat shock protein (HSP) is a potent inducer of natural and adaptive immunity, and the association between increased expression levels of HSPB1 and the risk of COPD has been reported and verified (Cui et al., 2015). The lipid repair enzyme GPX4, a member of the selenoprotein family, has been shown to act as a negative regulator of ferroptosis (Ji et al., 2022). Ferroptosis has an important role in the pathogenesis of COPD, and GPX4 expression levels were found to be significantly decreased in bronchial epithelial cells of COPD patients (Yoshida et al., 2019). FTL encodes ferritin light chain, which is involved in iron metabolism and utilization, and FTL protein can regulate ferroptosis by controlling free iron levels. FTH1 is responsible for intracellular iron storage and iron metabolism and altered expression of FTH1 is closely associated with the occurrence of ferroptosis. SOCS1 is a signal transducer of Janus kinase (JAK) and a negative regulator of the activator of transcription (STAT) pathway. Recent studies have found that SOCS1 is associated with the pathogenesis of COPD and is related to the time of COPD onset (Liao et al., 2022).

Based on these eight COPD macrophage ferroptosis-related genes, we performed co-expression analysis as well as drug prediction, and we identified a total of 121 COPD macrophage ferroptosis related DEGs that were significantly enriched in the MAPK signaling pathway. MAPK is a group of protein kinases that phosphorylate serine and threonine. Three signaling pathways are known, JNK1/2/3, REK1/2 and p38, which can regulate stress, apoptosis, immune defense and other biological processes. Recent studies have found that ferroptosis is closely related to the MAPK signaling pathway (Guo et al., 2022). In addition, MAPK signaling pathways has been shown to induce ferroptosis and to regulate the polarization of macrophages (Li et al., 2018). During the present study, significant reductions in M1 macrophages were observed, resulting in an imbalance in the polarization between M1 and M2 macrophages, which further exacerbates the pathology of the disease (Finicelli et al., 2022).

To better guide COPD treatment, we made drug predictions for these eight macrophage ferroptosis-related genes. In our study, insulin was predicted to target SOCS1 and treat COPD. As a common comorbidity of COPD, insulin resistance is mainly associated with smoking, obesity, genetics, lack of exercise, and long-term application of glucocorticoids, which can aggravate airway inflammation in COPD patients (Anker et al., 2022; Park et al., 2022). In addition, quercetin, which targets HSPB1, is a phenolic compound belonging to the flavonoid family and is found in various foods or agricultural products, such as onions, apples and broccoli, with high antioxidant activity (Li et al., 2022b). Quercetin can alleviate the course of COPD through various mechanisms, such as reducing lung inflammation and macrophage infiltration, and is expected to be one of the drugs for COPD (Araujo et al., 2022). Leukotrienes play an important role in the pathogenesis of COPD, and 5-LO can regulate the production of leukotrienes, while zileuton, an inhibitor of 5-LO, can significantly improve the exercise ability and quality of life in COPD exacerbation patients with a better safety profile (Garland et al., 2022). As a natural flavonoid present in a variety of herbs, lignan can reduce the expression of inflammatory factors and eliminate oxygen free radicals in LPS-induced mouse macrophage models, which may have therapeutic effects on COPD (Li et al., 2021). Since the efficacy of these drugs and targets is based solely on theoretical predictions, further animal experiments and clinical trials are required to provide more evidence.

Then, we further screened two biomarkers (HSPB1 and SOCS1) by multiple machine learning and built an ANN diagnostic model to evaluate their performance. The performance was assessed by AUC (0.816). Also, we testified the diagnostic ability in the validation set and the AUC was 0.777. Together, the developed diagnostic model could offer a novel perspective on our research of the mechanism of COPD.

In addition, we performed ROC analysis and external dataset validation, and performed single gene GSEA and GSVA analysis for biomarkers. HSPB1 expression was found to be closely related to the Hedgehog signaling pathway as well as glutathione metabolism. Recent studies suggest that Hedgehog signaling could be altered in COPD, promotes airway inflammatory processes and induces apoptosis in bronchial epithelial cells (Lahmar et al., 2022). Therefore, inhibition of Hedgehog signaling activation may serve as a new therapeutic. Glutathione is a major intracellular antioxidant that correlates with the severity of COPD. Glutathione levels are increased in bronchoalveolar lavage fluid of patients with stable COPD, whereas glutathione levels are reduced in patients with deteriorating COPD, suggesting that increasing intrapulmonary glutathione levels may be a new therapeutic strategy (Engelen et al., 2004). Further, SOCS1 is primarily involved in the folate biosynthesis signaling pathway, and folate levels were significantly decreased in patients with COPD (Kim et al., 2020).

We also identified upstream miRNAs and lncRNAs of those two biomarkers. We found that three of miRNAs (miR-34a-5p, miR-155–5p and miR-146a-5p) can target two biomarkers at the same time, so they are considered as key miRNAs (Figure 7). The miR-34a-5p has been found to be elevated in the airway epithelium of COPD patients (Zeng et al., 2022). In addition, miR-146a-5p in lung fibroblasts can inhibit IL-8 secretion, while miR-146a-5p expression is reduced in COPD patients, and the inhibitory effect on IL-8 secretion is diminished, thus exacerbating the development of chronic inflammation and COPD (Osei et al., 2017). Moreover, miR-155–5p is a classical multifunctional miRNA involved in the development, differentiation, activation, and maintenance of homeostasis of macrophages. For example, miR-155–5p expression was increased in alveolar macrophages of COPD mice (De Smet et al., 2020). Subsequently, we found that XIST (a key regulator of inflammatory response, that is significantly upregulated in lung tissue of COPD patients (Chen et al., 2021)) can simultaneously target three key miRNAs. And XIST is reported to negatively regulate the expression of miR-155–5p (Zhang et al., 2021; Wang and Cao, 2022). Moreover, SOCS1 and miR-155 are believed to be key regulators of the inflammatory response. The expression of miR-155 has also been demonstrated to be induced in animals by smoke, and miR-155 plays a role in the inflammatory response to lung injury by inhibiting the expression of SOCS1 (Zhang et al., 2020). Thus, we propose that the XIST/miR-155–5p/SOCS1 axis plays a significant role in the development and progression of COPD.

Despite the contribution of this study to the understanding of COPD macrophages, our study has several limitations due to small sample size and lack of information-rich sample data: first, the accuracy of COPD assessment and prediction could be improved by increasing the sample size; second, the macrophage biomarkers identified in this study with potential drugs and associated pathways need to be further validated to provide practical evidence for clinically targeted therapies; Third, analysis of protein expression levels of marker macrophages genes could provide substantial evidence. And the ferroptosis-induced cell death assay is necessary, some ferroptosis inhibitors or agonists may be used in experimental research to perfect this scientific problem. Despite recent advances in understanding, the relationship between macrophage ferroptosis and the HSPB1 and SOCS1 level needs to be further explored. In addition, the relationship between macrophage ferroptosis and chronic inflammation of COPD deserves to be investigated. Therefore, more extensive *in vivo* and *in vitro* experiments are necessary for further validation in the future.

## 5 Conclusion

In conclusion, we explored the two biomarkers (HSPB1 and SOCS1) of macrophage ferroptosis in COPD by combining scRNA-seq and RNA-seq with a view to treating COPD. Our study provides new theoretical insights into the role of macrophage ferroptosis biomarkers in COPD.

## Data Availability

The original contributions presented in the study are included in the article/supplementary material, further inquiries can be directed to the corresponding authors.
